# Angiopoietin-2 predicts morbidity in adults with Fontan physiology

**DOI:** 10.1038/s41598-019-54776-w

**Published:** 2019-12-04

**Authors:** Aditya S. Shirali, Gentian Lluri, Pierre J. Guihard, Miles B. Conrad, Helen Kim, Ludmila Pawlikowska, Kristina I. Boström, M. Luisa Iruela-Arispe, Jamil A. Aboulhosn

**Affiliations:** 1 Department of Surgery, University of California Los Angeles, Los Angeles, CA USA; 2 Ahmanson/UCLA Adult Congenital Heart Disease Center, Division of Cardiology, University of California Los Angeles, Los Angeles, CA USA; 3 Division of Cardiology, University of California Los Angeles, Los Angeles, CA USA; 4 Department of Radiology and Biomedical Imaging, University of California San Francisco, San Francisco, CA USA; 5 Center for Cerebrovascular Research, Department of Anesthesia and Perioperative Care, University of California San Francisco, San Francisco, CA USA; 6 Institute for Human Genetics, University of California San Francisco, San Francisco, CA USA; 7 Department of Molecular, Cell & Developmental Biology, Molecular Biology Institute and Jonsson Comprehensive Cancer Center, University of California Los Angeles, Los Angeles, CA USA

**Keywords:** Angiogenesis, Predictive markers

## Abstract

Morbidity in patients with single-ventricle Fontan circulation is common and includes arrhythmias, edema, and pulmonary arteriovenous malformations (PAVM) among others. We sought to identify biomarkers that may predict such complications. Twenty-five patients with Fontan physiology and 12 control patients with atrial septal defects (ASD) that underwent cardiac catheterization were included. Plasma was collected from the hepatic vein and superior vena cava and underwent protein profiling for a panel of 20 analytes involved in angiogenesis and endothelial dysfunction. Ten (40%) of Fontan patients had evidence of PAVM, eighteen (72%) had a history of arrhythmia, and five (20%) were actively in arrhythmia or had a recent arrhythmia. Angiopoietin-2 (Ang-2) was higher in Fontan patients (8,875.4 ± 3,336.9 pg/mL) versus the ASD group (1,663.6 ± 587.3 pg/mL, p < 0.0001). Ang-2 was higher in Fontan patients with active or recent arrhythmia (11,396.0 ± 3,457.7 vs 8,118.2 ± 2,795.1 pg/mL, p < 0.05). A threshold of 8,500 pg/mL gives Ang-2 a negative predictive value of 100% and positive predictive value of 42% in diagnosing recent arrhythmia. Ang-2 is elevated among adults with Fontan physiology. Ang-2 level is associated with active or recent arrhythmia, but was not found to be associated with PAVM.

## Introduction

Patients with functional single ventricle require a staged surgical approach starting in the neonatal period and eventually culminating in total cavopulmonary connection or atriopulmonary connection (Fontan palliation) typically during early childhood. The Fontan procedure, which functionally separates the systemic and pulmonary circulations, alleviates chronic volume overload and cyanosis at the expense of a variety of chronic end-organ injuries^1^. Morbidity of the Fontan procedure is common and includes ventricular dysfunction, rhythm and conduction disturbances, hepatomegaly, ascites, peripheral edema, protein losing enteropathy, and pulmonary arteriovenous malformations (PAVMs)^2^. While diagnosis of these complications is routine, early detection and prevention of these complications continues to challenge clinicians. The development of effective noninvasive strategies to detect and target these complications is essential for the treatment of this patient group. One such approach has been the identification of biomarkers to predict burden, prognosis, or potential response to a particular treatment of choice.

The use of biomarkers is increasingly being integrated into the management of adult congenital heart disease (CHD)^3^. As the number of Fontan patients reaching adulthood increases, a significant interest has emerged in utilizing biomarkers to predict Fontan failure and the development of PAVMs^4–8^. Elucidating the etiology of PAVMs in Fontan patients may pinpoint a potential noninvasive biomarker for the presence and severity of these malformations. Growing evidence suggests that the liver may play a role in the development of PAVMs. This is supported by the findings that the PAVMs are found in the lung or lungs that do not receive hepatic venous effluent, and redirection of hepatic blood flow to the pulmonary arteries leads to resolution of the PAVMs^9–11^. While several theories implicate the presence of an anti-angiogenic factor in hepatic venous effluent, no study has specifically examined the plasma protein profile of the hepatic vein in patients with PAVM to identify a putative factor responsible for PAVM development^12,13^.

In this study, we examined the molecular profile of the hepatic vein in Fontan patients with and without PAVMs to identify potential factors involved in arteriovenous malformation development. Furthermore, we used a similar approach to identify profile differences between Fontan patients and non-Fontan patients and proteins involved in the detection of Fontan morbidity.

## Results

### Patient characteristics

Clinical characteristics of patients with Fontan circulation (n = 25) and with atrial septal defects (ASD, n = 12) included in this study are shown in Table 1. Among adult congenital heart disease patients who undergo comprehensive diagnostic or interventional cardiac catheterization, patients with atrial septal defects are considered to have a simple congenital defect and most closely resemble patients with biventricular function. There was no statistical difference between the age of the Fontan group (32.2 ± 9.1 years) and the age of the ASD group (28.6 ± 10.6 years, p = 0.29). Forty percent of the Fontan group were females and 25% of the ASD group were males. Among the Fontan group the initial diagnoses included: 20% with dextro-transposition of great vessels (d-TGA), 20% with double outlet right ventricle, 16% with double inlet left ventricle, 16% with tricuspid atresia, and 28% labeled as ‘other,’ including hypoplastic left heart syndrome, unbalanced AV canal, and pulmonary atresia. Current Fontan types included lateral tunnel (52%), extracardiac (32%), and atriopulmonary (16%). Among these patients, 6 patients (3 extracardiac and 3 lateral tunnel) had a history of Fontan fenestration, among which 5 patients had the fenestration closed more than 8 years prior to study inclusion, and one patient with residual small fenestration at the time of cardiac catheterization. The average Fontan pressure during cardiac catheterization was 15.3 ± 5.0 mmHg.

**Table 1:** Baseline Demographic and Clinical Data of Study Group.

	Fontan (n = 25)	Atrial Septal Defect (n = 12)
Age, y	32.2 ± 9.1	28.6 ± 10.6
Male, n (%)	10 (40)	3 (25)
Diagnosis, n (%)		
Atrial Septal Defect	0 (0)	12 (100)
d-TGA	5 (20)	0
Double Outlet RV	5 (20)	0
Double Inlet LV	4 (16)	0
Tricuspid Atresia	4 (16)	0
Other	7 (28)	0
**Fontan Type, n (%)**		
Lateral Tunnel	13 (52)	—
Extracardiac	8 (32)	—
Atriopulmonary	4 (16)	—
Central Venous Pressure, mmHg	15.3 ± 4.9	7.6 ± 3.2
Fontan Pressure, mmHg	15.3 ± 5.0	—
Presence of AVM n (%)	10 (40)	0
History of PLE, n (%)	1 (4)	0
Clinical Ascites, n (%)	8 (32)	0
**Liver Biopsy n (%)**		
Normal	2 (8)	—
Fibrosis	6 (24)	—
Cirrhosis	4 (16)	—
Not Performed	13 (52)	12
Arrhythmia within 6 months, n (%)	5 (20)	0
History of Arrhythmia, n (%)	18 (72)	0
Atrial Flutter	10 (56)	—
Atrial Tachycardia	5 (28)	—
Other	2 (16)	—

d-TGA, dextro-Transposition of Great Vessels; LV, Left Ventricle; RV, Right Ventricle; AVM, arteriovenous malformation; PLE, Protein-Losing Enteropathy.

Forty percent of Fontan patients had evidence of PAVM at the time of plasma collection, while no ASD patients had PAVMs. One Fontan patient had a history of protein-losing enteropathy and 32% of Fontan patients had clinical evidence of ascites. Of the twenty five Fontan patients, twelve underwent liver biopsy which demonstrated normal tissue in two patients, fibrosis in six patients, and cirrhosis in four patients. Seventy-two percent of the Fontan patients had a history of arrhythmia, while none of patients in the ASD group had a history of arrhythmia. Among the Fontan group, five patients (20%) were either in active arrhythmia or had a history of arrhythmia within 6 months of plasma collection.

### Plasma analysis in Fontan patients

To identify a putative hepatic factor involved in PAVM development, blood was collected from the hepatic vein and superior vena cava and plasma protein levels from both locations were compared. We performed a Luminex Assay of plasma obtained from both locations in Fontan patients with AVM (n = 4), Fontan patients without AVM (n = 2), and ASD patients without PAVM (n = 6) to probe for 20 plasma proteins involved in angiogenesis and endothelial dysfunction and the difference in levels between the two locations was calculated for those with PAVM and those without PAVM. Results are summarized in Table 2. No statistically significant differences were found in any of the 20 plasma proteins assays when comparing those with and without PAVM, suggesting that the hepatic and systemic venous circulations are similar in regards to these specific plasma proteins. For this particular analysis, the number of Fontan patients is too small to separately analyze the different types of Fontan patients

**Table 2:** Differences in analyte concentrations (pg/mL) between Fontan and ASD patients.

Analyte (pg/mL)	Fontan (n = 6)	ASD (n = 6)	P-Value
Ang-2	7,710.3 ± 2,490.9	1,279.0 ± 637.8	0.000514447*
BMP9	454.0 ± 286.8	359.8 ± 348.6	0.653182
CD44	3,538.7 ± 2317.8	4,006.8 ± 2,614.8	0.772179
E-Selectin	34,278.2 ± 18,351.3	27,824.4 ± 13,118.6	0.54023
EGF	167.5 ± 182.6	129.3 ± 105.2	0.69593
Endostatin	37,581.1 ± 22,718.2	23,190.8 ± 7,085.4	0.213307
FABP4	22,240.5 ± 10,520.7	7,084.5 ± 3,491.6	0.0156468
FGF23	40.8 ± 30.6	11.2 ± 15.5	0.0899409
Gas6	12,481.9 ± 1,928.5	10,038.0 ± 1,721.7	0.0674726
ICAM1	364,736.7 ± 324,691.3	310,227.1 ± 229,749.8	0.767095
MCP-1	114.6 ± 27.3	94.6 ± 26.8	0.276038
MMP1	181.3 ± 99.3	120.9 ± 89.4	0.341724
MMP13	2,093.4 ± 1,229.5	1,863.5 ± 1,462.3	0.794634
MMP3	4,197.9 ± 1,867.9	3,398.9 ± 937.7	0.417549
MMP7	4,874.0 ± 3,775.1	1,212.2 ± 698.6	0.065511
MMP9	11,518.6 ± 2,993.2	10,956.7 ± 2,116.4	0.740628
Tenascin C	9,419.2 ± 1,739.3	7,548.6 ± 1,848.7	0.137993
TIMP1	60,061.3 ± 14,914.8	35,175.4 ± 4,833.5	0.0157517
Thrombospondin-2	59,903.9 ± 20,234.4	19,256.1 ± 16,455.9	0.00825944
vWF	3,418.5 ± 2,896.7	3,220.0 ± 1,781.3	0.899399

Ang-2, angiopoietin-2; BMP9, bone morphogenic protein 9; EGF, epidermal growth factor; FABP4, fatty acid binding protein 4; FGF23, fibroblast growth factor 23; Gas6, growth arrest-specfic protein 6; ICAM1, intracellular adhesion molecule 1; MCP-1, monocyte chemoattractant protein-1; MMP, matrix metalloproteinase; TIMP1, tissue inhibitor of metalloproteinase 1; vWF, von Willebrand factor.

**Figure 1:**
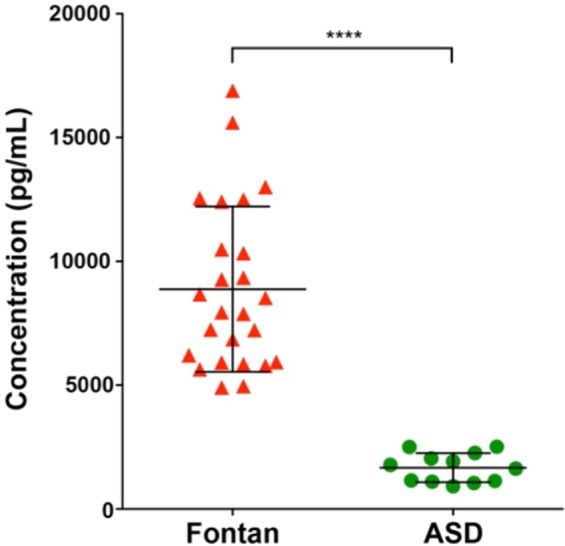
Plasma angiopoietin-2 levels in in Fontan patients (red triangle, n = 25) and ASD patients (green circle, n = 12). Angiopoietin-2 levels are significantly higher in Fontan patients (p < 0.0001). Lines represent mean ± standard deviation.

However, when comparing those with Fontan physiology (n = 6) to those with ASD (n = 6), differences that were statistically significant, as defined as p ≤ 0.0025 to account for multiple comparisons with the Bonferonni correction, were observed in Ang-2 (Table 2). Angiopoietin-2, a protein involved in the regulation of permeability, angiogenesis, and inflammation, was found to be 6-fold higher in patients with Fontan physiology than in ASD patients (p < 0.001)^14^. Further analysis of Ang-2 levels in the remaining cohort of patients showed a persistent elevation in Fontan patients when compared to ASD patients (Fig. 1).

**Figure 2:**
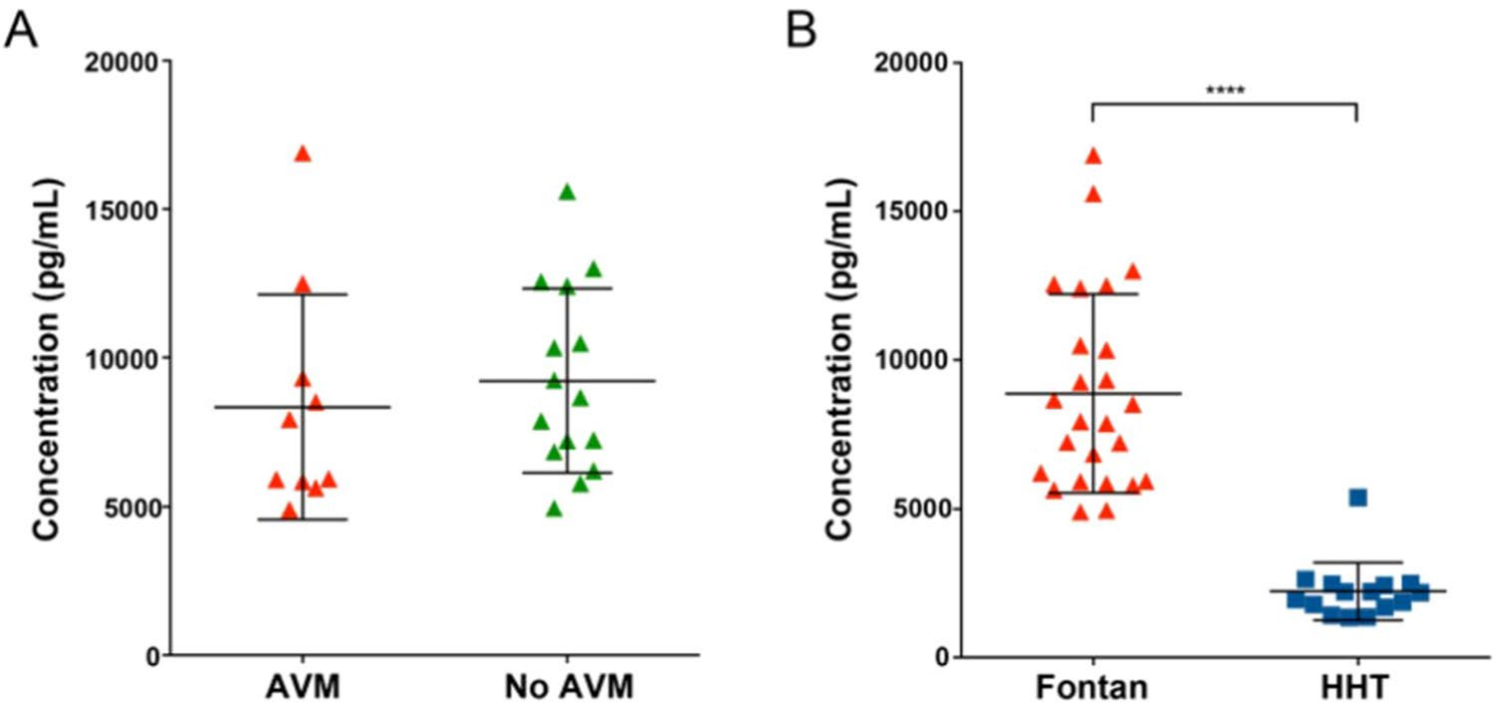
(**A**). Plasma angiopoietin-2 levels in in Fontan patients with AVM (red triangle, n = 10) and without AVM (green triangle, n = 15). There is no significant difference in angiopoietin-2 levels in Fontan patients with and without AVMs (p = 0.53) (**B**). Plasma angiopoietin-2 levels in in Fontan patients (red triangle, n = 25) and HHT patients (blue square, n = 23). Angiopoietin-2 levels are significantly higher in Fontan patients (p < 0.0001). Lines represent mean ± standard deviation.

Fontan patients had Ang-2 levels of 8,875.4 ± 3,336.9 pg/mL compared to ASD patients with 1,663.6 ± 587.3 pg/mL, a 5.3-fold difference (p < 0.0001). As seen in the Luminex Assay, Ang-2 has no correlation with PAVM formation and was confirmed in the entire cohort of Fontan patients. Angiopoietin-2 levels were 8,342.7 ± 3,780.1 pg/mL in Fontan patients with PAVM (n = 10) and 9,230.5 ± 3,092.8 pg/mL in those without PAVM (n = 15), demonstrating no statistical difference (p = 0.53, Fig. 2A). Furthermore, when comparing Ang-2 levels in Fontan patients to patients with hereditary hemorrhagic telangiectasia (HHT) and confirmed PAVMs, Ang-2 was found to be 4.2-fold higher in Fontan patients (8,875.4 ± 3,336.9 pg/mL) than in HHT patients with PAVMs (2,227.1 ± 968.5 pg/mL) as seen in Fig. 2B.

### Clinical outcomes

Given the absence of Ang-2 elevation in HHT patients with PAVM and the consistent elevation in all Fontan patients regardless of the presence of AVM, the elevation of this protein in Fontan patients likely serves as a marker of endothelial and cardiac dysfunction rather than an angiogenic marker. There was no statistically significant correlation between Ang-2 level and Fontan pressure measured during cardiac catheterization (*r* = 0.1599, p = 0.54). Fontan patients with clinical evidence of ascites had average Ang-2 levels of 10,339.6 ± 3,558.2 pg/mL versus those without ascites who had average levels of 8,186.3 ± 3,096.7 pg/mL (p = 0.14, Supplementary Figure 1A). In addition, no association between Ang-2 levels and degree of liver fibrosis or cirrhosis on liver biopsy was found (p = 0.14). The average Ang-2 levels in those with cirrhosis was 5,863 ± 797.7 pg/mL (n = 4) versus 8,475.5 ± 3,150.2 pg/mL (n = 6, p = 0.15) in those with fibrosis. Interestingly, Fontan patients with history of arrhythmia had average Ang-2 levels of 9,692.5 ± 3,379.2 pg/mL compared to those with no arrhythmia history with levels of 6,374.7 ± 1,651.0 pg/mL which trended toward statistical significance (p = 0.07). Those with active arrhythmia (defined as a clinically symptomatic arrhythmia that necessitated intervention by either medication adjustment or electrophysiology study/ablation) or arrhythmia within 6 months (20% of patients) had an average Ang-2 level of 11,396.0 ± 3,457.7 pg/mL versus those without a recent history with levels of 8,118.2 ± 2,795.1 pg/mL (p < 0.05). Forty-two percent of patients with levels above 8,500 pg/mL were in active arrhythmia or had a recent arrhythmia, while no patients with values less than 8,500 pg/mL were in active arrhythmia or had recent history of arrhythmia (p < 0.05, Supplementary Figure 1B). Using 8,500 pg/mL as a cutoff threshold, Ang-2 has a negative predictive value of 100% and suggests plasma Ang-2 levels may be used to screen for those Fontan patients at risk for development of an arrhythmia.

## Discussion

The present study tests the hypothesis that differences in plasma protein profiles exist between the hepatic vein and systemic venous circulation and that these differences account for the development of PAVM in Fontan patients. Multiple studies have demonstrated the formation of PAVMs whenever hepatic venous effluent does not perfuse the pulmonary arteries directly, either congenitally or postoperatively^10,15^, suggesting the presence of an anti-angiogenic factor in the hepatic effluent that when absent induces vascular proliferation and angiogenesis in the pulmonary bed^12^. To test this hypothesis, we examined the difference in concentrations of plasma proteins involved in inhibiting angiogenesis and endothelial dysfunction in the hepatic vein and superior vena cava in those with and without PAVMs. No differences were seen in anti-angiogenic proteins BMP9, Endostatin, Gas6, Thrombospondin-2, or TIMP1. Furthermore, no differences were seen in any of the 20 plasma proteins queried, suggesting similar protein profiles between the hepatic vein and the systemic venous circulation.

However, comparing the venous plasma proteins of Fontan patients with ASD patients shows demonstrable differences in several proteins involved in angiogenesis, Ang-2, FABP4, TIMP1, and Thrombospondin-2, with only Ang-2 meeting the criteria for statistical significance and the other three proteins approaching significance. Thrombospondin-2 and TIMP1 are both glycoproteins involved in inhibiting degradation of cell-matrix interactions and extracellular matrix, and have shown potent anti-angiogenic activities^16,17^. Elevations in these proteins in Fontan patients may reflect a systemic compensatory mechanism to the excessive pulmonary angiogenesis involved in PAVM formation. FABP4, a protein involved in VEGF-mediated endothelial cell proliferation and implicated in the development of cerebral cavernous malformations, was found to be 2.5–3 fold higher in Fontan patients compared to ASD patients^18^. However, on further analysis, no differences in FABP4 were detected between those with clinically evident PAVMs and those without PAVMs (data not shown). Angiopoietin-2 was the only plasma protein queried in the Luminex Analysis that was statistically significantly higher in Fontan patients than ASD patients, which was further validated with the entire cohort of patients enrolled.

Ang-2 is an endothelial cell specific protein that is rapidly released in response to various stimuli, including inflammatory cytokines, activated leukocytes, and hypoxia^19,20^. Release of Ang-2 induces loss of junctional integrity and inhibits endothelial cell quiescence, enhancing vascular permeability, inflammation and vascular remodeling^14,21^. It is especially pertinent that this protein is so elevated in Fontan patients who often exhibit excessive vascular permeability including presence of ascites, pleural effusions and protein losing enteropathy. In this regard, it is possible to speculate that the dysfunction in endothelial barrier is a consequence of excessive Ang-2. Nevertheless, we found no relationship between Ang-2 and PAVM formation both in Fontan patients and HHT patients, suggesting the role Ang-2 plays in Fontan physiology is likely unrelated to PAVM formation.

Apart from its involvement in tumor-specific angiogenesis, Ang-2’s role in inducing vascular permeability has led to evaluation of its role in critical illness, sepsis, volume overload, and cardiogenic shock^22–25^. Studies in critical care medicine have demonstrated that patients in septic shock exhibit remarkable levels of endothelial activation with release of Ang-2 resulting in acute lung injury, hepatic dysfunction, coagulopathy, and acute kidney injury^22,26^. In fact, these studies have identified a threshold of 5,900 pg/mL as being associated with higher mortality^26,27^. Furthermore, Ang-2 levels greater than 2,500 pg/mL were independent predictors of 28-day and 1-year mortality in patients with cardiogenic shock^28^. Interestingly, the Ang-2 levels in the Fontan patients included in this study were on average 4-fold higher than the levels predictive of increased mortality following cardiogenic shock and 1.5-fold higher than the levels associated with increased mortality in sepsis, highlighting the precarious long-term condition of Fontan patients and the need for close follow-up.

Yet, while all Fontan patients examined had Ang-2 levels greater than 2,500 pg/mL, and most had levels greater than 5,900 pg/mL, many of these patients had good functional capacity and led active lifestyles. Therefore, we examined whether the subgroup of Fontan patients with elevated Ang-2 levels may have more potent endothelial dysfunction and whether Ang-2 levels may be associated with and potentially predict complications related to Fontan failure, particularly ascites or arrhythmias. Although we did not observe an elevation in Ang-2 levels in those with ascites, we observed that patients with a history of arrhythmia had elevated Ang-2 levels that approached statistical significance. Studies have demonstrated that patients in chronic atrial fibrillation have elevated levels of plasma Ang-2^29,30^. As such, we performed subgroup analysis of the cohort of patients with an arrhythmia history and found that patients who were in active arrhythmia at the time of plasma collection or had a sustained arrhythmia within the previous 6 months had statistically higher levels of Ang-2 levels than those without recent arrhythmia. Furthermore, using 8,500 pg/mL as a threshold, we found that Ang-2 has a negative predictive value of 100% and a positive predictive value of 42% of active or recent arrhythmia. The fairly low positive predictive value is not unexpected, given Ang-2’s association with sepsis, cardiogenic shock, and angiogenesis^14,26,28^. However, the fact that no patients with levels less than 8,500 pg/mL had recent arrhythmias may be informative and may portend a more favorable prognosis in this population.

This study examines the association between a cohort of plasma proteins involved in angiogenesis and endothelial dysfunction with complications in Fontan patients, particularly PAVMs and arrhythmias. In the process, we identified Ang-2 as a potential indicator of disease burden, particularly in association with arrhythmias. However, this study is not without its limitations. We attempted to identify biomarkers related to PAVM formation in Fontan patients based on the theory of a putative hepatic factor that was first posited in patients with a superior cavopulmonary connection (SCPC), in which one or both lungs are devoid of hepatic vein effluent. Yet, significant regression of PAVMs has been noted after redistribution of flow with Fontan completion. Of note, we circumvented this redistribution of plasma proteins after Fontan completion by selectively obtaining plasma directly from the hepatic vein and performing multiplex analysis on a panel of proteins involved in angiogenesis and endothelial dysfunction. We found no significant differences between the panel of proteins in those without and without PAVMs. This outcome may be due to the biased selection of proteins in our panel or the existence of an undetectable or unselected protein; or it may indicate different etiological factors involved in PAVM formation in Fontan patients with no separation of hepatic and systemic venous circulation versus those with SCPC. The coincidental finding of Ang-2 elevation in Fontan patients and its association with critical illness is both compelling and concerning given the strong statistical difference found between Fontan patients in comparison to both ASD and HHT patients and the marked elevation in comparison to values that predict increased mortality. Nonetheless, longitudinal studies are necessary to fully validate the robustness of Ang-2 as a prognostic indicator of arrhythmia. Another limitation is that, for the control group our study does not include patients with other cardiovascular conditions that have elevated inferior vena cava pressures like RV dysfunction or severe tricuspid valve regurgitation.

In conclusion, we found no differences in hepatic plasma protein profile between those with and without PAVM. Plasma Ang-2 levels, however, were found to be significantly elevated in all Fontan patients. Ang-2 elevation was not found to predict PAVM formation, but we identified an association with active or recent arrhythmia. We believe our results warrant future longitudinal investigation to fully elucidate the prognostic potential of this endothelial activator.

## Methods

### Study sample, sample collection, and clinical assessment

Patients aged ≥18 years treated at the Ahmanson/UCLA Adult Congenital Heart Disease Center who had previously undergone a Fontan procedure and who also underwent routine cardiac catheterization between March 2016 and April 2017 at Ronald Reagan UCLA Medical Center were prospectively consented for study inclusion. Age- and sex-matched patients with atrial septal defects (ASDs) undergoing cardiac catheterization, selected based on the distribution of Fontan participants by sex and 10-year age group rather than matched to individual participants, were identified. The study was approved by the University of California, Los Angeles institutional review board (IRB) and informed consent was obtained prior to enrollment from all participants. All methods were performed in accordance with relevant guidelines and regulations.

Fontan patients underwent cardiac catheterization for assessment of Fontan hemodynamics and/or electrophysiology study if clinically indicated, while ASD patients underwent catheterization for percutaneous defect closure. During cardiac catheterization, blood was collected from the hepatic vein and superior vena cava. Presence of arteriovenous malformation (AVM) was determined by detection of five or more bubbles in the left atrium after injection of agitated saline into the distal right and left pulmonary arteries during transesophageal echocardiography.

Medical records were retrospectively reviewed for patient demographic information and clinical characteristics. Clinical information was collected pertaining to pre-Fontan diagnosis, Fontan type, presence of AVM, and Fontan-related clinical outcomes, including ascites, protein-losing enteropathy, liver cirrhosis/fibrosis and arrhythmia. From a prior IRB-approved study of AVM patients at University of California, San Francisco, fifteen venous plasma samples were obtained from patients with a diagnosis of hereditary hemorrhagic telangiectasia (HHT) by Curaçao criteria and PAVM screening and confirmation according to the International HHT Guidelines^31^.

### Biomarker assessment

Blood collected via cardiac catheterization was processed to plasma within 30 minutes to an hour of collection and frozen at −80 °C. A Luminex Screening Assay (R&D Systems Inc., Minneapolis, MN), a polystyrene bead-based multi-analyte profiling platform, was performed for twenty analytes involved in angiogenesis and endothelial dysfunction. This assay was performed on plasma samples from the hepatic vein and superior vena cava from twelve patients (six Fontan patients and six ASD patients). Angiopoietin-2 (Ang-2) concentration was measured with an enzyme-linked immunoassay on all plasma samples (R&D Systems Inc, Minneapolis, MN). The assessed inter-assay precision of the kit used demonstrated a coefficient of variation between 7.4 and 10.4%. Intra-assay precision demonstrated a coefficient of variation between 4.2 and 6.9%. Measurements were performed in duplicate at 1:5 dilutions. Measurement variability was minimal and comparable with that reported in the kit.

### Statistical analysis

Frequencies were tabulated for categorical variables. The two-sided unpaired Student *t* test was used to compare continuous variables. ANOVA was performed to evaluate differences in continuous variables between three or more groups. For comparison of analyte levels identified in the Luminex Assay, a Bonferonni correction for multiple comparison was used when determining the level of statistical significance, setting a p-value limit of 0.0025 for statistical significance. The Fisher exact test was used to analyze categorical variables between groups stratified by Ang-2 level with a p-value for statistical significance set at less than 0.05. A Pearson correlation was performed between Ang-2 level and continuous variables of interest. All analyses were performed using GraphPad Prism 6 (La Jolla, CA).

### Ethics approval

University of California Los Angeles IRB. University of California San Francisco IRB.

## Supplementary information

Supplementary Figure 1

## References

[1] Gewillig M, Brown SC (2016). The Fontan circulation after 45 years: update in physiology. Heart.

[2] Ohuchi H (2016). Adult patients with Fontan circulation: What we know and how to manage adults with Fontan circulation?. J Cardiol.

[3] Rajpal S, Alshawabkeh L, Opotowsky AR (2017). Current Role of Blood and Urine Biomarkers in the Clinical Care of Adults with Congenital Heart Disease. Curr Cardiol Rep.

[4] Opotowsky, A. R. *et al*. Galectin-3 Is Elevated and Associated With Adverse Outcomes in Patients With Single-Ventricle Fontan Circulation. *J Am Heart Assoc*, **5**, 10.1161/JAHA.115.002706 (2016). 10.1161/JAHA.115.002706 PMC4859390 26755550

[5] Marino BS (2017). Abnormalities in serum biomarkers correlate with lower cardiac index in the Fontan population. Cardiol Young.

[6] Wu FM (2017). Predictive value of biomarkers of hepatic fibrosis in adult Fontan patients. J Heart Lung Transplant.

[7] Kolcz J, Tomkiewicz-Pajak L, Wojcik E, Podolec P, Skalski J (2011). Prognostic significance and correlations of neurohumoral factors in early and late postoperative period after Fontan procedure. Interact Cardiovasc Thorac Surg.

[8] Field-Ridley A (2013). Endostatin, an inhibitor of angiogenesis, decreases after bidirectional superior cavopulmonary anastamosis. Pediatr Cardiol.

[9] Clement B, Musso O, Lietard J, Theret N (1999). Homeostatic control of angiogenesis: A newly identified function of the liver?. Hepatology.

[10] Srivastava D (1995). Hepatic venous blood and the development of pulmonary arteriovenous malformations in congenital heart disease. Circulation.

[11] AboulHosn J (2007). Regression of pulmonary arteriovenous malformations after transcatheter reconnection of the pulmonary arteries in patients with unidirectional Fontan. Congenit Heart Dis.

[12] Hoffman JI (2013). Normal and abnormal pulmonary arteriovenous shunting: occurrence and mechanisms. Cardiol Young.

[13] Duncan BW, Desai S (2003). Pulmonary arteriovenous malformations after cavopulmonary anastomosis. Ann Thorac Surg.

[14] Scholz A, Plate KH, Reiss Y (2015). Angiopoietin-2: a multifaceted cytokine that functions in both angiogenesis and inflammation. Ann N Y Acad Sci.

[15] Moore JW, Kirby WC, Madden WA, Gaither NS (1989). Development of pulmonary arteriovenous malformations after modified Fontan operations. J Thorac Cardiovasc Surg.

[16] Krady MM (2008). Thrombospondin-2 modulates extracellular matrix remodeling during physiological angiogenesis. Am J Pathol.

[17] Vandooren J (2015). Circular trimers of gelatinase B/matrix metalloproteinase-9 constitute a distinct population of functional enzyme molecules differentially regulated by tissue inhibitor of metalloproteinases-1. Biochem J.

[18] Cataltepe S, Arikan MC, Liang X, Smith TW, Cataltepe O (2015). Fatty acid binding protein 4 expression in cerebral vascular malformations: implications for vascular remodelling. Neuropathol Appl Neurobiol.

[19] Fiedler U (2006). Angiopoietin-2 sensitizes endothelial cells to TNF-alpha and has a crucial role in the induction of inflammation. Nat Med.

[20] Pichiule P, Chavez JC, LaManna JC (2004). Hypoxic regulation of angiopoietin-2 expression in endothelial cells. J Biol Chem.

[21] Saharinen P, Eklund L, Alitalo K (2017). Therapeutic targeting of the angiopoietin-TIE pathway. Nat Rev Drug Discov.

[22] Agrawal A (2013). Plasma angiopoietin-2 predicts the onset of acute lung injury in critically ill patients. Am J Respir Crit Care Med.

[23] Mikacenic C (2015). Biomarkers of Endothelial Activation Are Associated with Poor Outcome in Critical Illness. PLoS One.

[24] Tsai YC (2017). The interaction between fluid status and angiopoietin-2 in adverse renal outcomes of chronic kidney disease. PLoS One.

[25] Poss J (2015). Angiopoietin-2 in acute myocardial infarction complicated by cardiogenic shock–a biomarker substudy of the IABP-SHOCK II-Trial. Eur J Heart Fail.

[26] Fisher J (2016). Elevated Plasma Angiopoietin-2 Levels Are Associated With Fluid Overload, Organ Dysfunction, and Mortality in Human Septic Shock. Crit Care Med.

[27] Lukasz A (2008). Circulating angiopoietin-1 and angiopoietin-2 in critically ill patients: development and clinical application of two new immunoassays. Crit Care.

[28] Link A (2013). Circulating angiopoietins and cardiovascular mortality in cardiogenic shock. Eur Heart J.

[29] Freestone B, Chong AY, Lim HS, Blann A, Lip GY (2005). Angiogenic factors in atrial fibrillation: a possible role in thrombogenesis?. Ann Med.

[30] Choudhury A, Freestone B, Patel J, Lip GY (2007). Relationship of soluble CD40 ligand to vascular endothelial growth factor, angiopoietins, and tissue factor in atrial fibrillation: a link among platelet activation, angiogenesis, and thrombosis?. Chest.

[31] Faughnan ME (2011). International guidelines for the diagnosis and management of hereditary haemorrhagic telangiectasia. J Med Genet.

