# Supplementary material for: Angiopoietin-2 predicts morbidity in adults with Fontan physiology
Source: Sci Rep. 2019 Dec 4;9:18328. doi: 10.1038/s41598-019-54776-w (PMC6892891; doi:10.1038/s41598-019-54776-w)
Supplement: Supplementary file 1 — Supplementary Figure 1 [file 41598_2019_54776_MOESM1_ESM.pdf]

Angiopoietin-2 predicts morbidity in adults with Fontan physiology

Aditya S. Shirali [a‡], Gentian Lluri [b‡*], Pierre J. Guihard [c], Miles B. Conrad [d], Helen Kim [e,f], Ludmila Pawlikowska [e,f], Kristina I. Boström [c], M. Luisa Iruela-Arispe [g], Jamil A. Aboulhosn [b*]

[a] Department of Surgery, University of California Los Angeles, Los Angeles, CA
[b] Ahmanson/UCLA Adult Congenital Heart Disease Center, Division of Cardiology, University of California Los Angeles, Los Angeles, CA
[c] Division of Cardiology, University of California Los Angeles, Los Angeles, CA
[d] Department of Radiology and Biomedical Imaging, University of California San Francisco, San Francisco, CA
[e] Center for Cerebrovascular Research, Department of Anesthesia and Perioperative Care, University of California San Francisco, San Francisco, CA
[f] Institute for Human Genetics, University of California San Francisco, San Francisco, CA
[g] Department of Molecular, Cell & Developmental Biology, Molecular Biology Institute and Jonsson Comprehensive Cancer Center, University of California Los Angeles, Los Angeles, CA

[‡] These authors contributed equally to this work.

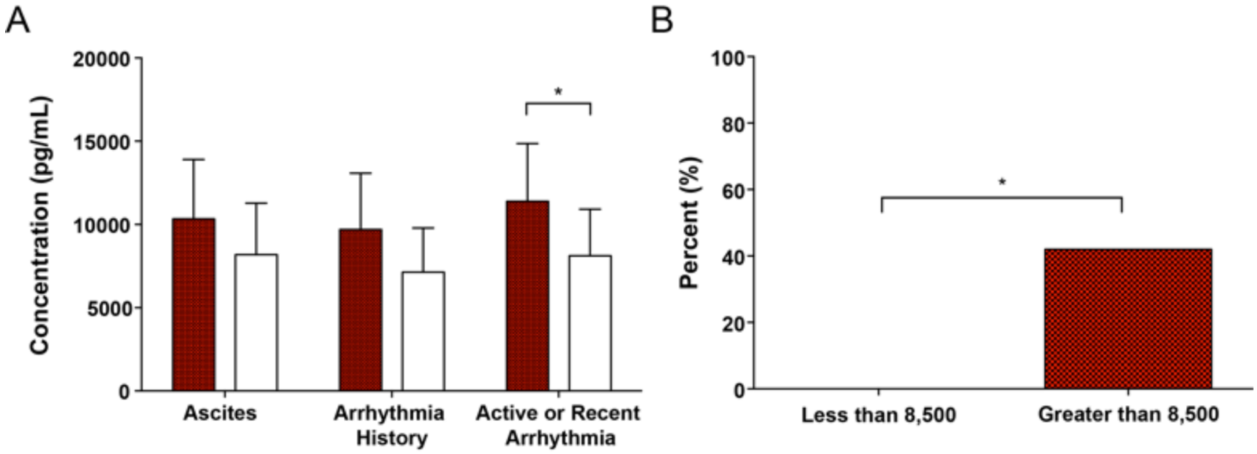

Supplementary Figure 1. A. Plasma angiopoietin-2 levels in patients with (red) and without (clear) ascites, history of arrhythmia and active or recent (within 6 months) arrhythmia. There is a significant difference in angiopoietin-2 in Fontan patients with active or recent arrhythmia ($p < 0.05$). B. The percent of Fontan patients with active arrhythmia or recent arrhythmia (within 6 months) with angiopoietin-2 levels greater than 8,500 pg/mL (n=5/12) and less than 8,500 pg/mL (n=0/13). A significant difference exists between these groups ($p < 0.05$)
